# Conservative Management of Placenta Accreta Using Helium Plasma Focused Radiofrequency Energy: A Surgical Technique

**DOI:** 10.7759/cureus.11832

**Published:** 2020-12-01

**Authors:** Bianca T Nguyen, Anthony Rodriguez, Naiya Patel, Diana Rodriguez, Peter Khamvongsa

**Affiliations:** 1 Obstetrics and Gynecology, Florida International University, Herbert Wertheim College of Medicine, Miami, USA

**Keywords:** morbidly adherent placenta, fertility, cesarean section, multiple cesarean section, cesarean hysterectomy, radio-frequency ablation, medical technology, obstetrics, placenta accreta, fulguration

## Abstract

The incidence of abnormal placentation has escalated due to the increase in cesarean sections. Adherent placentas are associated with significant maternal morbidity and mortality and often result in cesarean hysterectomy due to life-threatening hemorrhage. The purpose of these case reports is to describe conservative management of placenta accreta by utilizing a helium plasma device to fulgurate the placental bed. Placenta accreta is associated with a 7% mortality rate and 60% morbidity rate. Conservative treatment for uterine preservation include embolization, placenta left in-situ, uterine balloon tamponade, and methotrexate. Complications of these options include hemorrhage, endometritis, and morbidly adherent placentas (MAP) recurrence in subsequent pregnancies.

The helium plasma device utilizes radiofrequency (RF) to ionize helium into a plasma beam capable of coagulating and fulgurating tissue with high precision and minimal thermal spread. This instrument is Food and Drug Administration (FDA) approved for surgical coagulation and fulguration, but has not been evaluated in the treatment of placenta accreta at the time of a cesarean section.

The helium plasma device was used to fulgurate the placenta accreta at 40% power 4 L/min gas flow for 30 seconds, providing adequate hemostasis to the 12.76 cc of retained placental bed. Estimated blood loss was 560 cc. The patient remained hemodynamically stable and had no complications at follow up. The device provided efficient management of placenta accreta. This approach offers a safer alternative management of abnormal placentation and avoiding a cesarean hysterectomy. This novel surgical technique allows women with morbidly adherent placentas to maintain reproductive capability.

## Introduction

The incidence of morbidly adherent placentas (MAP) is steadily rising worldwide at an approximate rate of 1:500 deliveries and is correlated with the rising rates of cesarean deliveries [1]. On the spectrum of MAP, the least invasive is the placenta accreta that is an attachment of chorionic villi into the myometrium, the depth of which can be identified with advanced imaging [2]. Placenta accreta is associated with a 7% mortality rate and 60% morbidity rate [3].

MAP often results in a cesarean hysterectomy at the time of delivery due to uncontrolled and life-threatening hemorrhage [4,5]. There is no established screening protocol for MAP and those missed at antepartum appointments are typically diagnosed at the time of hemorrhage during placental extraction [5].

Imaging in the third trimester is vital to creating a plan before delivery [1]. Color Doppler ultrasound can assess the depth of infiltration into the myometrium. Doppler findings associated with accreta include loss of retro placental clear zone, presence of bridging vessels, and placental lacunae [6,7]. Studies have documented a sensitivity as high as 94% for the presence of intra-placental lacunae on Doppler for identifying cases of accreta [7]. A magnetic resonance imaging (MRI) can demonstrate the presence of accreta and the depth of invasion that can be particularly helpful for surgical planning of conservative methods. MRI without gadolinium in the second or third trimester can provide highly detailed images without the use of ionizing radiation [8]. The risks associated with an MRI with gadolinium may be outweighed by the significant maternal mortality associated with MAP. 

Conservative management of placenta accreta does not appear to compromise future fertility according to retrospective studies; however, the patient should be counseled that the risk of recurrence of MAP has been documented to be upwards of 30% [9].

Conservative methods for uterine preservation in placenta accreta include interventional radiology embolization, placenta left in-situ, uterine balloon tamponade, hemostatic suture techniques, methotrexate, and various forms of placental removal such as monopolar electrocautery [10-12]. Complications of these options include failure of uterine preservation, hemorrhage, endometritis, and recurrence of MAP in subsequent pregnancies as previously mentioned [9,11-14]. Other forms of electrosurgery have been utilized in managing placenta accreta, including monopolar energy [10-12].

The helium plasma device uses radiofrequency energy to ionize helium into a plasma beam capable of cutting, coagulating, and fulgurating tissue with high precision and little thermal spread [15-17]. This single user-friendly device works by creating cold plasma by transferring helium gas through an electrically charged surgical blade [15]. This electrosurgical tool is Food and Drug Administration (FDA) approved for open and laparoscopic surgery in a variety of surgical fields for fulguration, coagulation, and dissection. Uses in gynecologic procedures include transection of the round ligament, creation of the bladder flap, ovarian cystectomy, adhesiolysis, laparoscopic myomectomy, fulguration of endometriosis, and ablation of vulvar condylomas [17-19]. The precision of plasma energy allows for optimal depth of penetration with minimal injury to surrounding healthy tissue. The depth of fulguration, in millimeters, can reliably be selected by increasing or decreasing the power source. This device provides a safe and novel option to manage conservatively MAP to potentially minimize maternal morbidity and mortality and presents itself as a potential uterine and fertility-sparing option for patients.

A search of the literature revealed limited publications on the utilization of helium plasma radiofrequency (RF) energy for the treatment of placenta accreta during cesarean section. A case presented by Katsogiannou et al. attempted to utilize RF ablation to treat placenta accreta which had been left in situ days after delivery [20]. The patient suffered hemorrhagic episodes during and after delivery requiring red blood cell concentrate and fresh frozen plasma transfusions, uterine artery embolization, and subtotal hysterectomy [20]. As we used RF ablation at the time of and during the cesarean section, the timing may contribute to the effectiveness in the prevention of secondary complications for our patients. The authors do not describe the effectiveness of RF placental ablation during delivery.

## Case presentation

A 35-year-old gravida 2 para 1 female, with a history of one spontaneous abortion and one prior cesarean delivery in August 2016 presented to the clinic for prenatal care at 13-weeks gestation. Co-management with maternal-fetal medicine (MFM) was established due to advanced maternal age and a history of prior cesarean section. A transvaginal ultrasound performed by MFM during the twenty-third week of gestation revealed an area with increased vascularity near the prior cesarean section scar. A T2 MRI performed at 24 weeks gestation revealed an anterior and right lateral placenta with the anterior left inferior portion having a focal area of outpouching with secondary loss of the T2 low signal intensity. The MRI further confirmed increased vascularity in the region of the prior C-section scar tissue. The estimated placenta volume was 466.6 cc with 12.76 cc being abnormal placenta implantation (approximately 2.66% of the total placenta volume).

After reviewing the imaging results and discussing with the patient, cesarean delivery with the desire of uterine preservation was mutually agreed upon. Due to the precision and minimal energy spread, a helium plasma focused RF energy device was utilized to obtain hemostasis without damaging the healthy layers beneath the level of placental invasion. The patient was counseled on the risks of undergoing conservative treatment for MAP.

**Figure 1:**
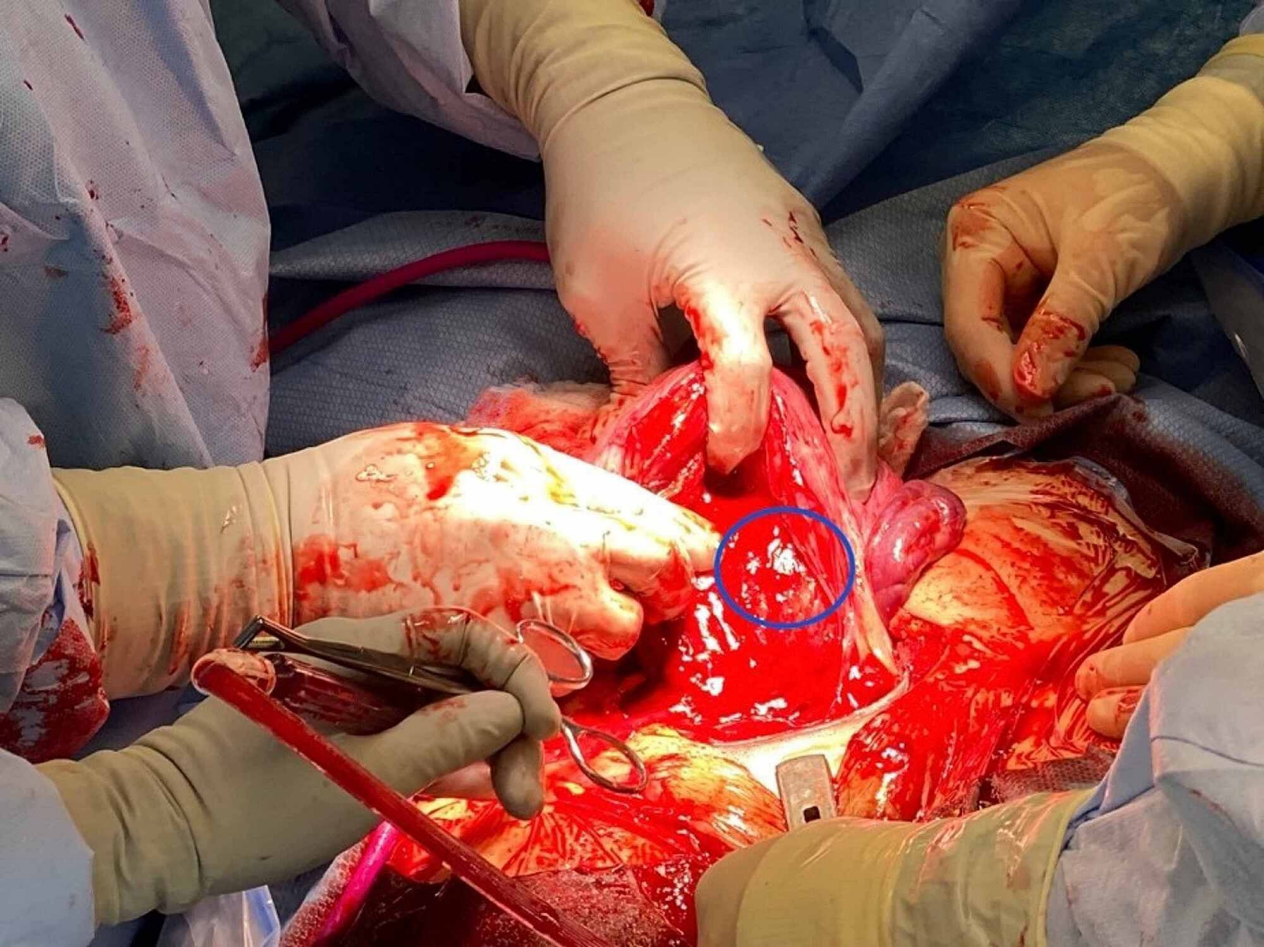
Site of placenta accreta prior to fulguration

**Figure 2:**
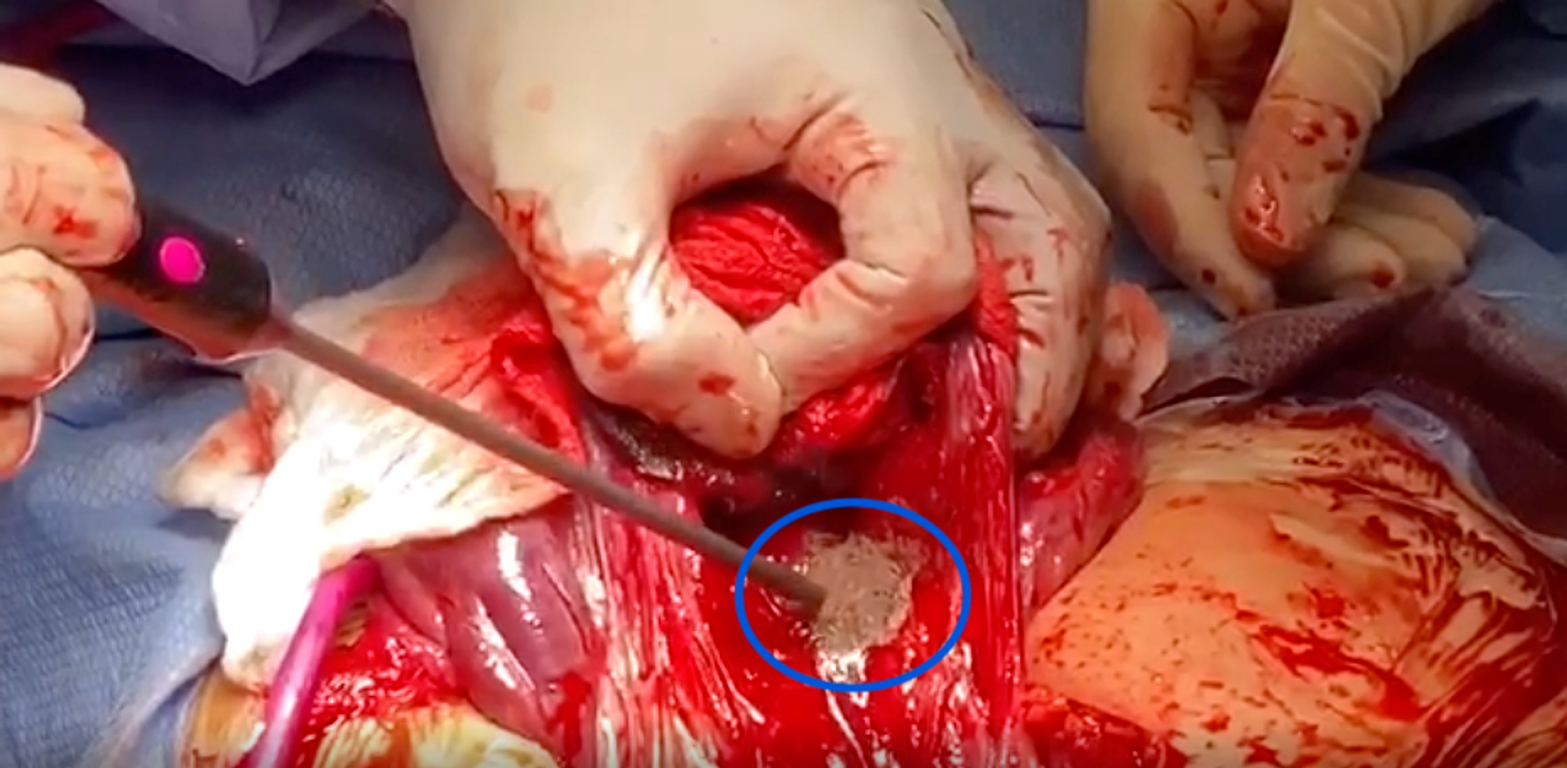
Placenta accreta after fulguration with the helium plasma device

On the day of cesarean delivery, manual extraction of part of the placenta was performed but was not successful due to adherent portions at the site of the prior c-section scar. The uterus was quickly exteriorized and retracted to achieve optimal visualization, as seen in Figure 1. The helium plasma RF device was used for 30 seconds on the endometrial surface to fulgurate the area of the retained placental tissue. The hand-held 5 mm diameter probe with a single push-button activator facilitated the mechanical and thermal energy via plasma flow to aid in the dissection of tissue [15]. While the standard device settings of 10% power and 4L/min are adequate to initiate energy, we utilized 40% power and 4 L/min for 25 seconds to increase the amount of heat and ensure effective treatment of the targeted tissue [15]. The light energy facilitated visualization of the target area, while the kinetic energy from heated helium plasma at the tip of the blade was employed to coagulate and fulgurate the uterine tissue [15]. A radius of 3 cm was created, and hemostasis was confirmed visually. Successful hemostasis was achieved with the thermal effect of helium plasma RF energy, as visualized in Figure 2.

Carboprost 0.25 mg was administered locally at the site of accreta to maintain uterine contraction and prevent further bleeding. The uterine incision was approximated and closed in a horizontal mattress fashion and the uterus was replaced into the pelvis. The patient was transferred to the post-anesthesia care unit for close monitoring. The quantitative blood loss was 560 cc. The preoperative hemoglobin of 10.5 g/dL decreased to 8.9 g/dL within 24 hours postoperatively. The patient remained hemodynamically stable and was discharged on postoperative day two. Blood transfusions and supportive care were not required. She did not develop endometritis and had no complications at the six-month follow-up. 

We present another patient case of a 35-year-old gravida 3 para 2 female with two prior cesarean sections who presented at six weeks and two days of gestation with a low-lying placenta. MFM consultation was requested was established, and the patient completed two visits throughout the pregnancy. A biophysical profile without stress test was performed at 27 weeks and three days due to vaginal bleeding, revealing a placenta with rounded configuration, concerning for a circumvallate placenta. The biophysical profile (BPP) score was 8/8. Evaluation of the partial placenta accreta was performed by MRI at 30 weeks and three days. The placenta was found to be anterior/right lateral, extending to the fundus and with a thickness of 4.1 to 5.0 cm in AP. The signal intensity of the placenta was intermediate on the T2 weighted images and demonstrated increased vascularity in the retro-placental space. Towards the upper and right lateral portion of the placenta, there was a questionable focal small area of loss of the retro-placental vascular space as well as the loss of the thin T2 dark uteroplacental interface. The findings were indicative of a focal area of placenta accreta.

The patient underwent a repeat cesarean section with a low transverse incision at 38 weeks and six days gestation with fulguration of the partial placenta accreta with the helium plasma device. On the day of surgery, the placenta was manually extracted and oxytocin was administered. Although most of the placenta was removed, a small area of placenta accreta on the middle aspect of the anterior uterus was identified. Methylergometrine and carboprost were administered at the placenta accreta site. The helium plasma technology was utilized at 40% power at 4L/minute to fulgurate the remaining tissue on the endometrium. An additional 0.25 mg of carboprost was administered to the area of fulguration. Quantitative blood loss was 450 cc. Pre-operative hemoglobin was 13.6 g/dL and post-operative hemoglobin was 12.7 g/dL. The placental pathology report revealed a placental disc weight of 391 grams. The singleton placenta measured 15.2 x 14.7 x 2.8 cm, with an eccentrically located 3-vessel umbilical cord without abnormalities. The patient remained hemodynamically stable, without postoperative complications.

## Discussion

Plasma medicine has improved in recent years, but the wide uses for this device have yet to be explored and approved in clinical care in the management of placenta accreta. The incidence of placenta accreta has been steadily rising presumably related to the increase in cesarean section rates and advancing maternal age [2,10] which will require more innovative and conservative techniques for management to combat the associated morbidity and mortality [4,14]. Conservative uterine-sparing methods have been employed in the past, although the development of MAP protocols is essential in improving maternal and fetal health [10]. This approach minimizes the need for blood transfusions or other invasive and morbid procedures.

This technology is versatile in its ability to employ mechanical, kinetic, thermal, and light energy to target areas of the placenta accreta [15]. After the depth of invasion is identified with advanced imaging, the device can be set to fulgurate at the depth desired to ensure the invasive tissue is targeted without damaging the underlying tissue. As there is increased accuracy in the depth of using this energy source, this device allows for the fulguration of ectopic placental tissue into the myometrium while controlling bleeding without the involvement of deeper tissue once hemostasis is achieved. The area of implantation was adequately cauterized with the helium plasma device, and methylergometrine and carboprost were supplemented to add uterine contractility and maintain stability. When compared with monopolar energy, argon beam coagulation, and carbon dioxide laser devices on porcine tissue at the same settings, the helium plasma device achieved lower lateral and depth of thermal spread [15]. The multi-modal capabilities of the device prevent the surgeon from switching tools mid-surgery which may shorten the time of the procedure [15]. The device does not require the use of eye protection and produces small amounts of smoke allowing for better visualization of the surgical field [15]. The safety for use of this device in gynecologic surgeries has been documented and studies demonstrate a superior safety profile for helium plasma energy in comparison to monopolar energy that has been previously utilized in the management of placenta accreta [12, 18,19]. Therefore, its heating and cooling properties make it a safe and high-precision method that minimizes collateral damage to surrounding healthy tissue.

## Conclusions

Obstetric hemorrhage and peripartum hysterectomy were avoided in our patients, allowing for future pregnancies. To our knowledge, this is the first documented case of utilizing a helium plasma device for safe and effective treatment of placenta accreta. Further research is necessary to investigate the safety and surgical application of the helium plasma tool in the treatment of abnormal placentation. While the helium plasma device is an FDA-approved device for open and laparoscopic surgeries, use in morbidly adherent placentation is not an FDA approved use of this tool. The helium plasma device is a viable option for the management of a focal placenta accreta to minimize maternal morbidity and mortality.
